# MASLD and MASH increase fracture risk in humans and mice by arresting new bone formation

**DOI:** 10.21203/rs.3.rs-7775325/v1

**Published:** 2025-11-24

**Authors:** Galen Goldscheitter, Vinay Jahagirdar, Mulugeta Seneshaw, Faridoddin Mirshahi, Morgan Summerlin, Allison Ip, Austin Coelho, Evan Buettmann, Damian Genetos, Arun Sanyal, Henry Donahue

**Affiliations:** Virginia Commonwealth University School of Medicine; Virginia Commonwealth University School of Medicine; Virginia Commonwealth University School of Medicine; Div. of Gastroenterology, Hepatology and Nutrition, Dept. of Internal Medicine, Virginia Commonwealth University School of Medicine, Richmond, VA; Georgia Institute of Technology; University of Virginia; Yale University; Virginia Commonwealth University; University of California Davis College of Veterinary Medicine; Virginia Commonwealth University; Virginia Commonwealth University College of Engineering

**Keywords:** MASLD, fracture, inflammation, skeletal metabolism

## Abstract

Early data suggest metabolic dysfunction-associated steatotic liver disease (MASLD) associates with increased fractures. However, absolute risk, subpopulations at greatest risk, and risk basis are unknown. We use a two-pronged approach to address these gaps: we investigated fracture risk among humans with MASLD and mechanisms among diet-induced animal model of NAFLD (DIAMOND^™^) mice. We interrogated the TriNetX US collaborative database, propensity-matching people with MASLD 10-fold with people with metabolic dysfunction alone. All-fracture and pathological-fracture risks are elevated among people > 51 years of age with MASLD. DIAMOND mice with MASLD lost bone thickness, strength, and bone formation and gained increased bone resorption. MASLD associated with differential expression of key indicators of bone loss: decreased hepatic *igf1* and *cyp2r1*, increased hepatic *fgf21, ctgf*, and *anxa2*, decreased skeletal *bglap, runx2*, and *postn*, and increased skeletal *pparg*. These expression changes are supported by increased serum FGF21, reported in literature to impair bone anabolism. Herein, we establish MASLD as a risk factor for fracture and propose putative mechanisms driving bone loss.

## INTRODUCTION

Metabolic dysfunction-associated steatotic liver disease (MASLD) is the most prevalent chronic liver disease globally and affects about a third of the adult population [1]. It is closely associated with underlying obesity, hypertension, type 2 diabetes (T2DM) and dyslipidemia, at least one of which is required for its diagnosis [2]. While much is known about hepatic and classical metabolic outcomes such as cardiovascular events [3], the spectrum of other outcomes remains relatively understudied.

Recent studies of patients with MASLD noted an increased number of fractures compared to healthy controls[4], [5], [6], [7], [8]. However, given the common presence of underlying metabolic dysfunction [2], it is unclear if the increase in risk is linked to liver disease or metabolic dysfunction. Further, these data have not been verified in large scale studies, and it is not known if there are specific populations such as women vs men, older adults, those with alcohol or nicotine use, and persons stratified by ethnic groups have increased risk. Fracture risk is also linked to age [9]; the interaction of age and MASLD in the risk of fractures also remains unknown. Finally, the status of bone health, i.e. its strength, mineralization, and its cellular and biochemical basis in MASLD remain unclear.

The MASLD population is aging, whichis associated with increased fracture risk [9]. Further, recently approved drugs from MASLD such as incretin memetics, including glucagon-like peptide-1 (GLP-1) analogs and drugs in development such as fibroblast growth factor 21 (FGF21) analogs carry a potential risk of inducing bone loss [10]. Fractures, especially in older individuals, have a major adverse impact on their ability to function and manage activities of daily living [11]. For all these reasons, it is important to better elucidate the relationship of MASLD with bone health and fracture risk.

To address these unmet needs, we took a two-pronged approach. First, we interrogated the TriNetX US collaborative database which has anonymized electronic medical record (EMR) data from multiple health systems and covers over 100 million persons. We identified those with MASLD and propensity-matched them 10-fold to a control group with metabolic dysfunction but without known MASLD. We related the fracture profile across groups to assess the impact of age, biological sex, ethnicity, and alcohol and nicotine use on these relationships. Next, to obtain mechanistic insights on the bone itself, we used the diet-induced animal model of non-alcoholic fatty liver disease (DIAMOND^™^) which has been validated against human disease [12], [13]. In this model, we evaluated MASLD severity histologically and hepatic gene expression of factors known to affect bone health. We directly studied bone morphology, strength, cell populations, and skeletal gene expression to obtain mechanistic insights on the relationship between MASLD and bone. The relationship between fracture and MASLD among persons with metabolic dysfunction and proposed cellular and molecular mechanisms in mice are described below.

## MATERIALS & METHODS

### Fracture risk among people with MASLD

We conducted a retrospective cohort study using the TriNetX U.S. Collaborative Network, a federated database of de-identified EMRs from 65 participating healthcare organizations. We identified adult patients (> 18 years) with a diagnosis of MASLD, defined by the presence of hepatic steatosis in the context of overweight/obesity, diabetes mellitus, or other metabolic risk factors. Patients with evidence of advanced fibrosis or cirrhosis were excluded (Supp. Table 1). The MASLD cohort was restricted to those with concomitant prediabetes or T2DM. A control cohort was constructed of individuals with prediabetes or T2DM but without MASLD. Index date was defined as the earliest recorded diagnosis of MASLD, prediabetes, or T2DM during the study period. Patients were followed until the occurrence of a fracture, death, or last available encounter. The primary outcome was the incidence of any fracture. Secondary outcomes included osteoporosis-related fractures (hip, vertebral, or wrist), pathological fractures, and age-stratified fracture incidence. Outcomes were identified using validated ICD-10-CM diagnosis codes excluded (Supp. Table 1). Baseline covariates included age, sex, race, menopausal status, nicotine and alcohol use, glucocorticoid exposure, and loop diuretic exposure. These variables were selected based on prior evidence of association with fracture risk. Descriptive statistics were used to summarize baseline characteristics. Incidence of fractures was compared between cohorts using chi-square testing. Cox proportional hazards regression was performed to estimate hazard ratios (HRs) and 95% confidence intervals (CIs) for fracture risk associated with MASLD. Models were adjusted for demographic and clinical covariates. Age-stratified analyses were conducted in prespecified decade intervals (< 40, 40–49, 50–59, 60–69, 70–79, > 80 years). Analyses were performed using TriNetX’s embedded analytics platform. Virginia Commonwealth University does not consider TriNetX queries human subjects research. This study is therefore exempt from IRB review.

### Animal Studies

Twenty eight-week old male DIAMOND mice, a well-established animal model of MASLD [12], were randomized to chow diet (CD; Teklad 7012) and normal water (NW; from vivarium supply), or high-fat diet (HFD; Teklad 88137, 42% kcal from fat with 0.1% cholesterol) and sugar water (SW; 18.9 g/dL d-glucose, 23.1 g/dL d-fructose). Mice were fed *ad libitum* and housed on a 12h light-12h dark cycle in a 21–23°C vivarium. Cohorts of animals were humanely euthanized by CO_2_ asphyxiation 36 weeks following diet randomization, consistent with the development of MASH without the development of spontaneous hepatocellular carcinoma. All animal care procedures were performed according to protocols approved by the Virginia Commonwealth University Institutional Animal Care and Use Committee (IACUC AD10001341).

### Animal sample collection and processing

Mice were weighed and exposed to inhalant isoflurane anesthesia prior to humane euthanasia via cervical dislocation. A laparotomy was performed, the abdominal skin and muscle layers were dissected away, the peritoneal fascia parted, and the liver localized. The portal triad and inferior vena cava were resected, and the liver was removed *in toto*. Portions of liver were preserved in 10% neutral buffered formalin (NBF) for histologic processing or RNALater (ThermoFisher AM7020) for RNA assays. The skin overlying the patellar surface was then incised, the gastrocnemius and soleus muscles carefully isolated and preserved in RNALater, and femurs and tibias isolated and stored. One femur was fixed, with muscle in place, in 10% NBF for histology. The other femur was stored in RNALater for RNA assays. One tibia was wrapped in PBS-soaked gauze for mechanical testing via 3-point bending, and the other tibia was stored in RNALater as a backup sample for RNA assays. The remaining tissues were frozen at −80°C for future investigations.

### Micro-computed tomography

Femurs and tibias were scanned in 1% agar *ex vivo* on a Bruker 1276 benchtop micro-computed tomography (μCT) scanner using a 0.5 Al filter with 200 kV and 60 μA X-ray tube potential and current—respectively—and 730 ms integration time. Isotropic voxel sizes were 7 μm for femurs and 10 μm for tibias. Reconstruction, segmentation, and analysis were performed using Bruker software (NRecon, Dataviewer, CTAn). Analysis of cortical bone was performed in the mid-diaphysis. Cortical bone regions of interest (ROIs) were 180 μm long and established at the midpoint of the femur (26 slices). Tibias were analyzed only at the fracture site occurring in 3-point bending, and the outcomes were limited to the distance to the point of principal stress and moment of inertia for calculation of mechanical testing parameters. Cortical bone contouring was performed automatically in CTAn. Outcomes for cortical bone analysis included mean tissue area (T.Ar), bone area (B.Ar), bone area fraction (B.Ar/T.Ar), cortical thickness (Ct.Th), and tissue mineral density (TMD). Trabecular bone was analyzed in the metaphysis. Metaphyseal ROIs were created with an offset (2% of total bone length) proximal to the epiphyseal plate. The metaphyseal ROI length was 10% of the total bone length in both femurs and tibias. Trabecular bone was contoured manually in both metaphysis. All contours were drawn and quantified by a blinded user. Trabecular bone outcomes were tissue volume (TV), bone volume (BV), bone volume fraction (BV/TV), trabecular number (Tb.N), trabecular thickness (Tb.Th), trabecular spacing (Tb.Sp), and tissue mineral density (TMD).

### Assessment of bone mechanical properties

Tibias were broken in 3-point bending after μCT scanning on a Bose Electroforce 3200 with a 100 lbf load cell for force data capture. Tibias were placed on supports (10 mm spacing) with the apex of primary curvature at the midpoint, oriented with the anteromedial surface in tension. A mover was lowered until it contacted the posterolateral surface and a 0.5 N preload was applied. The mover was driven downwards at 1 mm/min until the bone fractured. Displacement and force data were recorded at 10 Hz. The distance between the proximal end of the tibia and the fracture site were recorded. This location was used as a reference to calculate moment of inertia and identify principal strain site on μCT. Outcomes for 3-point bending included ultimate load, ultimate stress, stiffness, Young’s modulus, work to fracture, and total toughness.

### Quantitative reverse transcription polymerase chain reaction (qRT-PCR)

Tissues stored in RNALater were homogenized using a bead mill (ThermoFisher 15-340-164) before RNA was isolated using RNeasy Micro Kits (Qiagen 74004). Total RNA concentration and 260/280 ratio were measured using a NanoDrop Lite spectrophotometer (ThermoFisher NDNDLUSCAN). Total RNA was reverse-transcribed into complementary DNA using iScript reverse transcriptase and random primers (BioRad 1708891). Quantification of the complementary DNA templates was performed by real-time PCR using SYBR green fluorescence (BioRad C1000 Touch thermocycler [1851148], BioRad CFX96 optical reaction module [1845097]). Primer pairs were as follows: *igf1* (BioRad, qMmuCED0044388), *ctgf* (BioRad qMmuCED0003632), *anxa2* (BioRad qMmuCID0005752), *fgf21* (BioRad qMmuCED0061148), *bglap* (BioRad qMmuCED0041364), *tnfsf11* (BioRad qMmuCID0026078), *postn* (BioRad qMmuCID0026147), *runx2* (BioRad qMmuCED0049270), actb* (BioRad qMmuCED0027505), and ywhaz* (BioRad qMmuCED0027504) (*housekeeping genes). Genes were selected for analysis based on biological significance and the sequencing results from our recent paper [14].

### Histologic sample processing

Femurs and livers were isolated and fixed in 10% NBF (24 hours, 4°C). Livers were immediately processed and embedded in paraffin. Femurs, however, were decalcified (14% ethylenediaminetetraacetic acid (EDTA), pH = 7.2, 14 days, 4°C) prior to processing and paraffin embedding. 5 μm-thick axial liver sections and sagittal bone sections were cut and mounted on positively-charged slides. All samples were heated (1 hour, 56°C) prior to deparaffinization and staining. Image analysis of histologic specimens was performed by blinded evaluators (QuPath v0.6.0) [15].

### Histologic MASLD severity scoring

From each mouse, one liver section was stained with hematoxylin and eosin (H&E) and one section was stained with picro-sirius red (PSR). Severity of steatohepatitis was evaluated on H&E-stained liver sections and quantified using the NAFLD activity score (NAS) algorithm [16]. Steatosis was scored as the percentage of hepatocytes containing fat droplets with the following cutoffs: 0 (< 5%), 1 (5–33%), 2 (33–66%), or 3 (> 66%). Lobular inflammation was scored as the number of inflammatory foci within a high-power field: 0 (no foci), 1 (< 2 foci), 2 (2–4 foci), or 3 (> 4 foci). Hepatocyte ballooning was quantified as 0 (none), 1 (few rare but definite cases of ballooning), or 2 (most hepatocytes with definite ballooning). Total NAS is the sum of each component. Fibrosis was scored on PSR-stained sections using the following staging algorithm: F0 (no fibrosis), F1a (mild, zone 3, perisinusoidal fibrosis), F1b (moderate, zone 3, perisinusoidal fibrosis), F1c (periportal fibrosis without accompanying perisinusoidal fibrosis), F2 (perisinusoidal and periportal fibrosis), F3 (bridging fibrosis), or F4 (cirrhosis).

### Osteoblast number & mineralizing surface

Osteoblast number (Ob.N) and mineralizing surface (MS) were assessed on H&E-stained femur sections, prepared as described above. A 1 mm segment of cortical bone at the mid-diaphysis was defined corresponding to the μCT region of interest at the mid-diaphysis. Osteoblasts were defined as cuboidal cells on the periosteal surface, most commonly occurring in groups adjacent to osteoclasts. Osteoblast number was manually counted and normalized to the total periosteal bone surface (BS). The mineralizing surface was measured as the surface length covered by osteoblasts and normalized to BS.

### Osteoclast number & surface

Osteoclast number and surface were assessed using tartrate-resistant acid phosphatase-stained femur sections. A 1 mm region of interest (ROI) was established corresponding to our cortical CT analysis region at the mid diaphysis. Osteoclasts were defined as TRAP^+^, multinuclear cells attached to bone surfaces within the ROI. Osteoclast number (Oc.N) was counted manually and the surface of each associated resorption pit (Oc.S) was outlined manually. Oc.N and Oc.S were each normalized to BS.

### Enzymatically-linked immunosorptive assay (ELISA)

Blood was collected via cardiac puncture at sacrifice. Samples were allowed to coagulate for 2 hours at room temperature. Serum was isolated via centrifugation and stored at −80°C. FGF21 levels were measured in serum via ELISA according to manufacturer protocol (BioTechne MF2100).

### Statistical Analysis

Intergroup differences in human subjects were assessed using a Cox proportional hazards model on the TriNetX Live online platform as described above. Intergroup differences between DIAMOND mice fed CD/NW or HFD/SW were assessed using an unpaired Students’ t-test in cases where outcomes were normally distributed and homoscedastic. Otherwise, intergroup differences were assessed via Mann-Whitney rank sum test. Normality of residuals in each outcome was tested via the Anderson-Darling method, and homoscedasticity via the Bartlett test. Outlier testing was performed using the iterative generalized extreme studentized deviate method in cases of normally distributed outcomes. In non-normal outcomes, outliers were defined as point more than 1.5 interquartile ranges above the 75th percentile, or below the 25th percentile. For all comparisons, we defined α = 0.05 and ß=0.20. Assessment of intergroup differences in DIAMOND mice was performed using GraphPad Prism (version 10.5.0), normality, variance, and outlier testing was performed in MATLAB (version R2024a).

## RESULTS

### Fractures are more common among persons with metabolic dysfunction and MASLD than those with metabolic dysfunction but no MASLD

**Figure 1:**
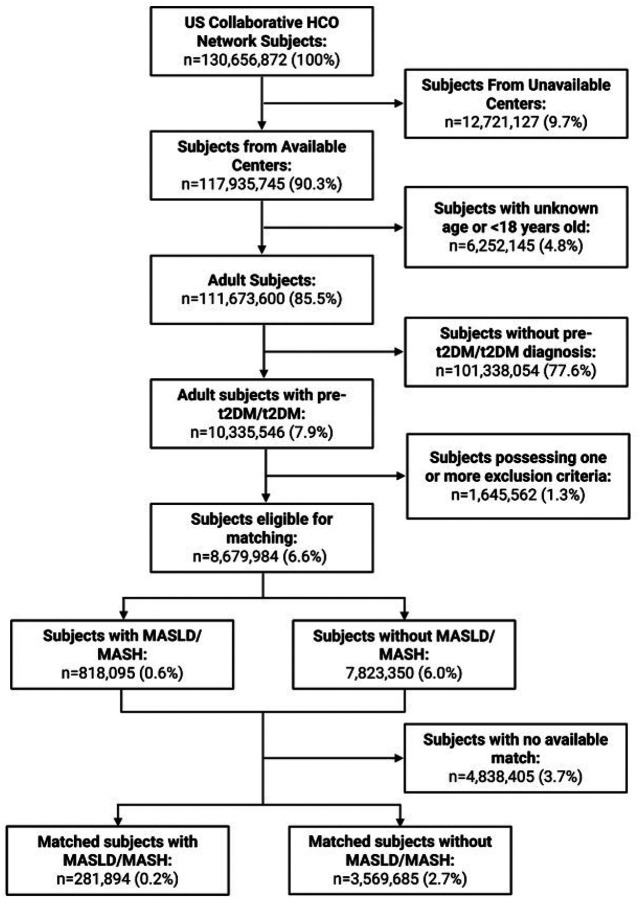
Participant inclusion and matching flowchart. Participant number (percent of available participants)

Among our cohort of 3,851,579 persons with metabolic dysfunction—defined as adults (≥18 years old) with previously diagnosed pre-type II diabetes mellitus or T2DM—281,894 people with MASLD/MASH were matched to 3,569,685 people without MASLD/MASH (Fig. 1). Among people with MASLD, a Cox proportional hazards model identified a significant increase in risk of fracture compared to those without (Table 1, HR=1.228, *p*<0.0001). Covariates for this model included sex, age, menopause status, nicotine use, alcohol use, glucocorticoid use, loop diuretic use, and ethnicity (**Supp. Table 1**). Notably, men were relatively protected from fracture (Table 1, HR=0.728, *p*<0.0001). Examining subsets separated by age deciles, people 41 years of age or older have increased risk of all-cause fracture and osteoporotic fracture, pathological fracture risk is elevated among people 51 years of age or older. These changes indicate that metabolic dysfunction, alone, does not explain skeletal fragility among people with MASLD and the liver disease is an independent risk factor for fracture.

### Male DIAMOND on HFD/SW become obese, develop MASH, and develop a hepatic transcriptome consistent with low bone masss

**Figure 2:**
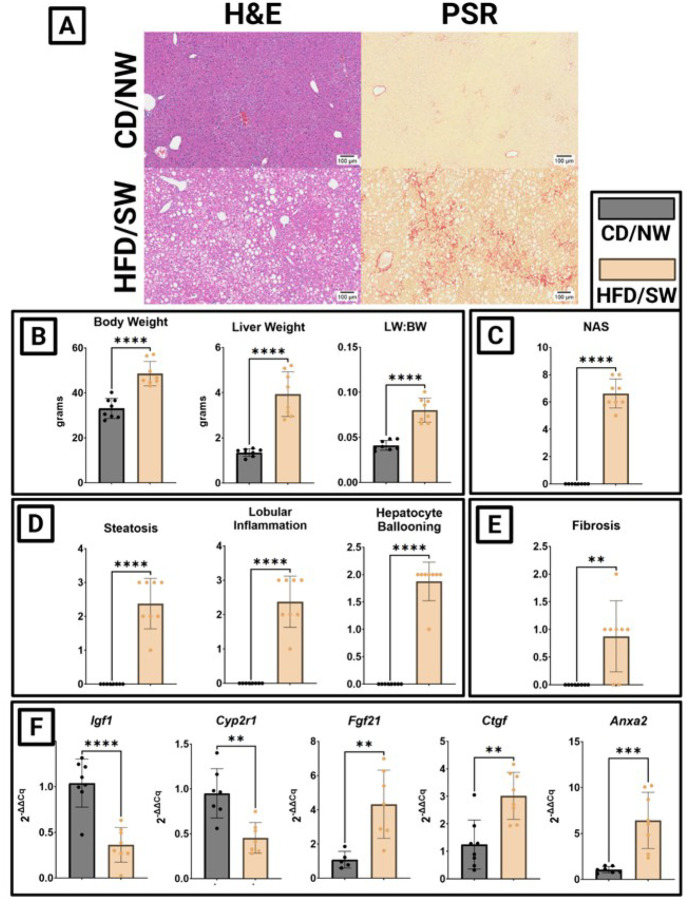
**Figure 3:** DIAMOND mice on HFD/SW develop MASH, lose hepatic expression of pro-bone forming genes and increase expression of bone-destroying genes. (A) H&E and picro-Sirius red stain of liver from DIAMOND mice. (B) Body, liver, and relative liver:body weights. (C) NAFLD activity score. (D) Individual NAS component scores. (E) Fibrosis score. (F) Expression of bone forming (igf1, cyp2r1) and bone-destroying (fgf21, ctgf, anxa2) genes. (*p<0.05, **p<0.01, ***p<0.001, ****p<0.0001)

After 36 weeks on HFD/SW, male DIAMOND mice developed severe hepatic steatosis and fibrosis (Fig. 2A). HFD/SW feeding resulted in increases in body weight and hepatomegaly (Fig. 2B). Livers from mice on HFD/SW were significantly larger than mice fed CD/NW. This increase in liver weight was not accounted for by compensatory hypertrophy secondary to increased body weight (Fig. 2B). The development of MASLD and/or MASH was assessed using NAS (NAFLD Activity Score) and Fibrosis Score. NAS increased significantly among mice fed HFD/SW compared to CD/NW controls (Fig. 2C). The subsets of NAS—steatosis, lobular inflammation, and hepatocyte ballooning—were individually significantly increased (Fig. 2D). Stage 1A or 1B perisinusoidal fibrosis developed among most DIAMOND mice, with one instance of stage 2 fibrosis. This NAS and fibrosis staging is consistent with the development of MASH (Fig. 2E). MASH associated with changes in hepatic gene expression consistent with a pro-bone resorption secretory phenotype. Hepatic expression of *igf1* and *cyp2r1* decreased and increased hepatic expression of *fgf21, ctgf*, and *anxa2* increased among HFD/SW mice versus CD/NW mice (Fig. 2F). Among male DIAMOND mice, obesity, hepatomegaly, and MASLD associate with loss in bone anabolic factors from liver (*igf1, cyp2r1*) and increased expression of suppressors of bone formation (*fgf21, ctgf, anxa2*).

### MASH drives cortical thinning and weakness without trabecular bone loss in DIAMOND mice

**Figure 3:**
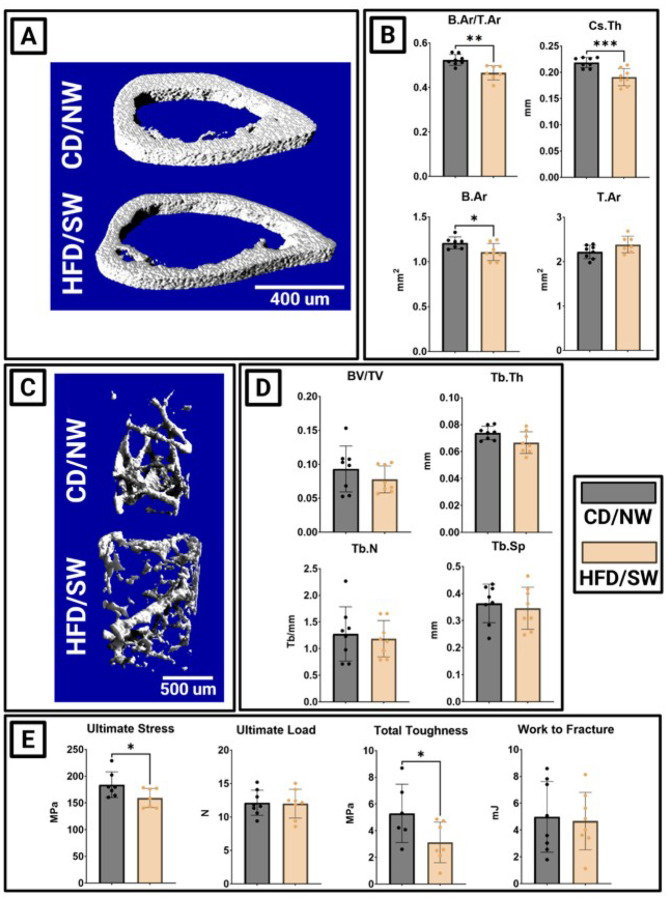
**Figure 4:** DIAMOND mice with MASH develop cortical thinning and weakness. (A) 3D reconstructions of DIAMOND mice femur cortical bone. (B) Cortical bone parameters: bone area per tissue area (B.Ar/T.Ar), cross-sectional thickness (Cs.Th). (C) 3D reconstructions of DIAMOND mice trabecular bone. (D) Trabecular bone parameters: bone volume per tissue volume (BV/TV), trabecular thickness (Tb.Th), trabecular number (Tb.N), and trabecular spacing (Tb.Sp). (E) Tibial strength from 3-point bending. (*p<0.05, **p<0.01, ***p<0.001)

Male DIAMOND mice with MASH lost cortical bone in the mid-diaphysis (Fig. 3A). MASH associated with decreased bone area fraction (B.Ar/T.Ar), cortical thinning, decreased bone area (B.Ar) that was not compensated for by decreased tissue area (T.Ar) (Fig. 3B). Indeed, while not statistically significant (p=0.07) the mean tissue area is greater among mice fed HFD/SW, suggesting expansion of the bone perimeter with a simultaneous decrease in the amount of bone present (Fig. 3B). DIAMOND mice with MASH lost no trabecular bone compared to healthy controls (Fig. 3C). Each trabecular bone index (BV/TV, Tb.Th, Tb.N, Tb.Sp) was preserved (Fig. 3D). Directly testing the mechanical integrity of bones, MASH associated with a decrease in ultimate stress and total toughness in 3-point bending, while ultimate load and work to fracture were preserved (Fig. 3E). These changes indicate cortical expansion with reduced endosteal formation, consistent with periosteal drift without compensatory osteogenesis.

### MASH associates with low bone-formation in DIAMOND mice

**Figure 4:**
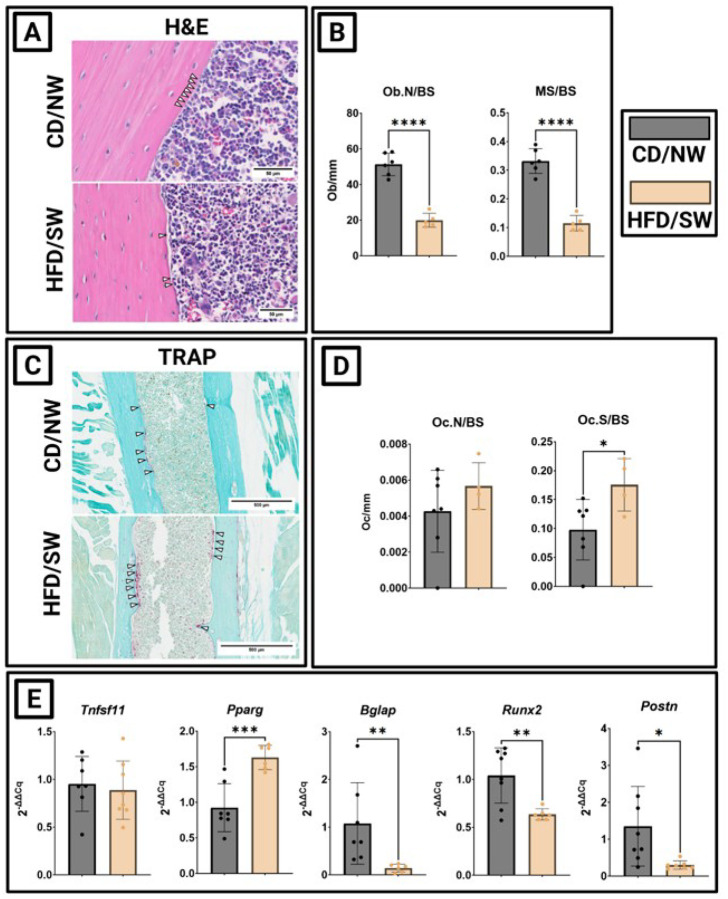
**Figure 5:** DIAMOND mice with MASH form fewer osteoblasts, more osteoclasts, and express deleterious changes in bone genes downstream of changes seen in liver. (A) H&E staining of femur. (B) Osteoblast parameters: osteoblast number per bone surface (Ob.N/BS), mineralizing surface per bone surface (MS/BS). (C) TRAP staining of femur. (D) Osteoclast parameters: osteoclast number per bone surface (Oc.N/BS), osteoclast surface per bone surface (Oc.S/BS). (E) Bone gene expression associated with bone resorption (tnfsf11, pparg) and formation (bglap, runx2, postn). (*p<0.05, **p<0.01, ***p<0.001, ****p<0.0001)

Male DIAMOND mice on HFD/SW developed fewer osteoblasts within their cortical bone than those on CD/NW (Fig. 4A). Their osteoblast density (Ob.N/BS) decreased, alongside decreased mineralizing surface relative to total cortical bone surface (MS/BS) (Fig. 4B). Osteoclast formation increased modestly, relative to the decrease in osteoblasts, among mice fed HFD/SW versus CD/NW counterparts (Fig. 4C). Their osteoclast density (Oc.N/BS) did not change, however, a modest increase was observed in osteoclast surface density (Oc.S/BS) (Fig. 4D). Differential gene expressions of key bone metabolism indicators suggested a failure of bone formation with bone resorption largely preserved among mice fed HFD/SW. Namely, bone *tnfsf11* expression did not change, *pparg* expression increased and *bglap, runx2*, and *postn* expression decreased (Fig. 4E). These changes indicate a failure of new bone formation without compensatory slowing of bone resorption—instead—a modest increase in bone resorption occurred.

## DISCUSSION

In recent decades, increased fracture risk and bone loss have been considered a likely, severe consequence of the growing population of people with MASH. Herein we show, in humans, that MASLD is an independent risk factor for bone fracture. This is supported by several meta-analyses that suggest MASH associates with decreased bone mineral density in both children and adults [17], [18] and that MASH increases fracture risk [5], [8]. This effect is age-dependent, as people with MASLD have increased fracture risk beyond the age of 41 years of age, particularly pathological fracture after 51 years of age. The magnitude of this effect is preserved or increases with age, suggesting it parallels or accelerates age-associated bone loss. Our human data were supported by our mouse data, which exhibit bone loss characterized by cortical thinning. Among bone tissue types, cortical bone contributes the most to overall strength. Thus, loss of cortical bone is thought to correlate with fracture risk. Thus, we hypothesize the increase in fracture risk in patients in MASLD and MASH may be due to specifically cortical bone loss. Future studies using human CT data would be necessary to support this hypothesis.

The relationship between MASLD and bone highlights significant bone and liver crosstalk which negatively impacts bone. In bone, we report a low bone formation phenotype characterized by decreased osteoblast number and activity with a concomitant–if mild–increase in osteoclast activity. This low bone formation phenotype appears to be driven by growth-inhibiting signaling from the liver characterized by reductions in *igf1* and *cyp2r1* in mice with MASLD vs controls. Insulin-like growth factor-1 (IGF-1, encoded by *igf1*) is critical to bone formation, regulating skeletal development, morphology and strength [19], [20]. Previous models of liver-specific IGF-1 KO demonstrated cortical bone loss without loss of trabecular bone [21], which recapitulates our findings of thinning and volume loss in cortical bone without trabecular bone loss. *Cyp2r1* encodes a critical vitamin D_3_ 25-hydroxylase (cytochrome P450 2R1, CYP2R1), whose expression correlates with circulating 25(OH)D_3_ [22]. In our MASH mice, *cyp2r1* expression fell 55% compared to healthy controls. Decreased hepatic *cyp2r1* likely drives lower circulating 25(OH)D_3_ and therefore impairs bone formation. Supporting the skeletal significance of decreased hepatic *igf1* and *cyp2r1*, osteoblast density within bone cortex and expression of bone formation markers fall in mice with MASH. In mice with MASH, the density of bone-forming osteoblasts is substantially lower than those without MASH. Concomitant with the drop in osteoblast density, *bglap, runx2*, and *postn* expression are decreased in bone indicating a relative osteoblast deficit, and therefore less bone formation. We hypothesize that decreased hepatic *igf1* and *cyp2r1* are in part responsible for this bone formation defect.

While MASLD seems to drive loss of bone anabolic factors from the liver, there is also induction of bone suppressive factors. In our mice, we observe increased hepatic *fgf21* (encodes FGF21) expression. The potential impact of hepatic *fgf21* expression is supported by increased serum FGF21, which would mediate its impact on bone. FGF21 and its analogues increase insulin sensitivity, reduce hepatic steatosis, and have antifibrotic activity, and thus are excellent drug candidates in MASLD [23]. However, FGF-21 induction of PPARγ (peroxisome proliferator-activated receptor gamma) is concerning for bone loss [24]. Consequently, we also see an increase in bone *pparg* (encodes PPARγ) expression, whose expression is observed to be induced by FGF-21 [25]. coinciding with deleterious changes in skeletal morphology by uCT and strength by 3PB. Within bone, PPARγ increases sclerostin expression among osteocytes, slowing new bone formation by inhibiting the Wnt-β-catenin pathway [26], [27]. The increase in bone *pparg* expression we observed, therefore, is likely to suppress new bone formation via alterations in osteocyte-mediated regulation of bone metabolism. The relationship between hepatic *fgf21*, skeletal *pparg*, and bone loss in the setting of MASH warrants further investigation.

Limitations in the human arm of this study include its retrospective nature and lack of stratification by MASLD severity or date-of-diagnosis. A large-scale, long-term prospective surveillance study is necessary to address these limitations. The major limitation in the mouse arm of this study is the use of male mice alone. We focused solely on male mice because—in our previous work—we observed no skeletal changes in female DIAMOND mice with MASH [14]. Female DIAMOND mice develop MASH when fed HFD/SW, however they develop milder liver disease. This may be due to a protective effect of estrogens [28]. Contrary to observations in our mice, in human subjects, we observe men with MASLD/MASH to be relatively protected against bone loss. This may be due to the age association with human fracture risk and MASLD. Fracture risk with MASLD is increased after 40 years of age. The average age of onset for menopause is 52 years of age [29]. Accordingly, women in the 50–80-year range likely have very little to no estrogen production, leading to a loss of the protective effects of estrogen in MASLD. As human men do not undergo an equivalent andropause, there may be low levels of estrogen signaling in men from aromatization of testosterone to estrogens which protect men relative to women of the same age. A study of ovariectomy in female mice would clarify the role of estrogens in MASLD fracture risk.

MASLD, and MASH, are prevalent diseases affecting over a third of the global population. Their annual incidence is increasing, and no cure is on the horizon. With fracture and bone loss recognized as comorbidities of MASLD and MASH, addressing the pathogenesis of bone disease in this setting is critical to alleviate morbidity, mortality, and global socioeconomic burden. Mechanisms for bone disease in MASLD have been proposed but experimental evidence is lacking. Several drug classes under evaluation, or recently approved, for the treatment of MASLD, may increase fracture risk, including thiazolidinediones, glucagon-like peptide analogs, and FGF-21 analogs. Establishing these mechanisms is needed to assess the fracture safety profile of these drugs in MASLD. In this study, we propose MASLD results in the failure of new bone formation, leading to fragility and fracture. We identify decreased hepatic *igf1* and *cyp2r1* expression, key proteins supporting bone formation, and increased hepatic *ctgf, fgf21*, and *anxa2* expression, inhibitors of bone formation, in the setting of MASH and bone loss. Future work should address these hepatic mediators of bone loss to attenuate the global disease burden of MASLD and MASH.

## Supplementary Material

Supplementary Files

This is a list of supplementary files associated with this preprint. Click to download.

- SUPPLEMENTALINFORMATION.docx

## Figures and Tables

**Table 1:** Cox regression model of all fracture hazard ratio (HR) among persons with MASLD vs no MASLD.

Factor	Hazard Ratio (95% CI)	*p*
MASLD	1.228 (1.213–1.244)	<0.0001
Male sex	0.728 (0.723–0.734)	<0.0001
Age	1.027 (1.027–1.027)	<0.0001
Menopause status	1.041 (1.025–1.057)	<0.0001
Nicotine dependence	1.412 (1.393–1.430)	<0.0001
Alcohol use	1.451 (1.249–1.686)	<0.0001
Glucocorticoid use	1.217 (1.207–1.227)	<0.0001
Loop diuretic use	1.249 (1.235–1.263)	<0.0001
Ethnicity, Caucasian	1.254 (1.243–1.266)	<0.0001
Ethnicity, Black/African American	0.808 (0.798–0.819)	<0.0001

**Table 2:** Fracture HR among persons with MASLD vs no MASLD, grouped by age deciles and fracture subtype.

Age, years	All fracture HR (95% CI)	Osteoporotic fracture HR (95% CI)	Cathological fracture HR (95% CI)
20–30	1.066 (0.872–1.302) *p* = 0.534	1.282 (0.823–1.997) *p* = 0.272	1.802 (0.589–5.514) *p* = 0.295
31–40	1.083 (0.966–1.215) *p* = 0.172	1.164 (0.094–1.490) *p* = 0.228	1.268 (0.709–2.265) *p* = 0.422
41–50	1.134 (1.059–1.213) *p* < 0.0001	1.195 (1.022–1.398) *p* = 0.026	1.231 (0.864–1.755) *p* = 0.249
51–60	1.101 (1.052–1.152) *p* <0.0001	1.248 (1.156–1.348) *p* < 0.0001	1.528 (1.268–1.843) *p* < 0.0001
61–70	1.128 (1.090–1.168) *p* < 0.0001	1.195 (1.148–1.243) *p* < 0.0001	1.314 (1.160–1.489) *p* < 0.0001
71–80	1.221 (1.180–1.264) *p* < 0.0001	1.188 (1.150–1.226) *p* < 0.0001	1.420 (1.265–1.595) *p* < 0.0001

## Abbreviations

MASLD: metabolic dysfunction-associated steatotic liver disease
MASH: metabolic dysfunction-associated steatohepatitis
T2DM: type 2 diabetes mellitus
FGF21: fibroblast growth factor 21
EMR: electronic medical record
DIAMOND: diet-induced animal model of non-alcoholic fatty liver disease
NAFLD: non-alcoholic fatty liver disease
ICD-10-CM: international classification of disease 10 clinical modification
HR: hazard ratio
CI: confidence interval
CD: chow diet
NW: normal water
HFD: high-fat diet
SW: sugar water
NAS: NAFLD activity score
μCT: micro-computed tomography
Ct.Th: cortical thickness
TMD: tissue mineral density
B.Ar: bone area
T.Ar: tissue area
BV: bone volume
TV: tissue volume,Tb.Th,trabecular thickness
Tb.N: trabecular number
Tb.Sp: trabecular spacing
EDTA: ethylenediaminetetraacetic acid
H&E: hematoxylin & eosin
PSR: picro-sirius red
Ob.N: osteoblast number
MS: mineralizing surface
BS: bone surface
Oc.N: osteoclast number Oc.S, osteoclast surface
BS: bone surface
EDTA: ethylenediaminetetraacetic acid
ROI: region of interest
IGF-1: insulin-like growth factor-1
CYP2R1: cytochrome P450 2R1
PPARG: peroxisome proliferator-activated receptor gamma
